# Eyelid bags

**DOI:** 10.1186/1746-160X-6-9

**Published:** 2010-06-18

**Authors:** Francesco Inchingolo, Marco Tatullo, Fabio M Abenavoli, Massimo Marrelli, Alessio D Inchingolo, Roberto Corelli, Angelo M Inchingolo, Gianna Dipalma

**Affiliations:** 1 Department of Dental Sciences and Surgery, University of Bari, Bari, Italy; 2 Department of Medical Biochemistry, Medical Biology and Physics, University of Bari, Bari, Italy; 3 Department of "Head and Neck Deseases", Hospital "Fatebenefratelli", Rome, Italy; 4 Department of Maxillofacial Surgery, Calabrodental, Crotone, Italy; 5 Department of Maxillofacial Surgery, University of Bari, Bari, Italy; 6 Department of Surgical, Reconstructive and Diagnostic Sciences, University of Milano, Milano, Italy

## Abstract

Eyelid bags are considered a sign of ageing, but they often appear prematurely due to the variety of causes that favor them. This brief report describes the case of a patient who was referred to us for the correction of a second degree bilateral palpebral ptosis that the patient had suffered from for several years and that in recent months had worsened to the point of interfering with vision and who, aside from modest eyelid bags, presented a massive protrusion of "preocular" fatty tissue. Despite the indication of classic blepharoplasty through a lower lid incision and, therefore, the possibility of removing excess skin, the patient opted instead only for the removal of the bulging fat. The patient's postoperative results were normal and the patient was extremely satisfied with both the correction of the ptosis and the "rejuvenating" effect of removing the protruding orbital fat in the eyelid.

## Introduction

Eyelid bags are considered a sign of ageing, but they often appear prematurely due to the variety of causes that favor them. A survey conducted by Goldberg. et al. [1] highlights how the presence of bulging orbital fat tissue is the last cause of this condition for a high percentage of patients. There are obviously a variety of causative pathophysiologic mechanisms and even if some of these are not shared by all authors, there is no doubt that a slackening of orbicular muscle tension, lower lid horizontal laxity also secondary to a weakness of canthal support and the weakening of the orbital septum all favor a prolapse of orbital fat.

## Case Report

**Figure 1:**
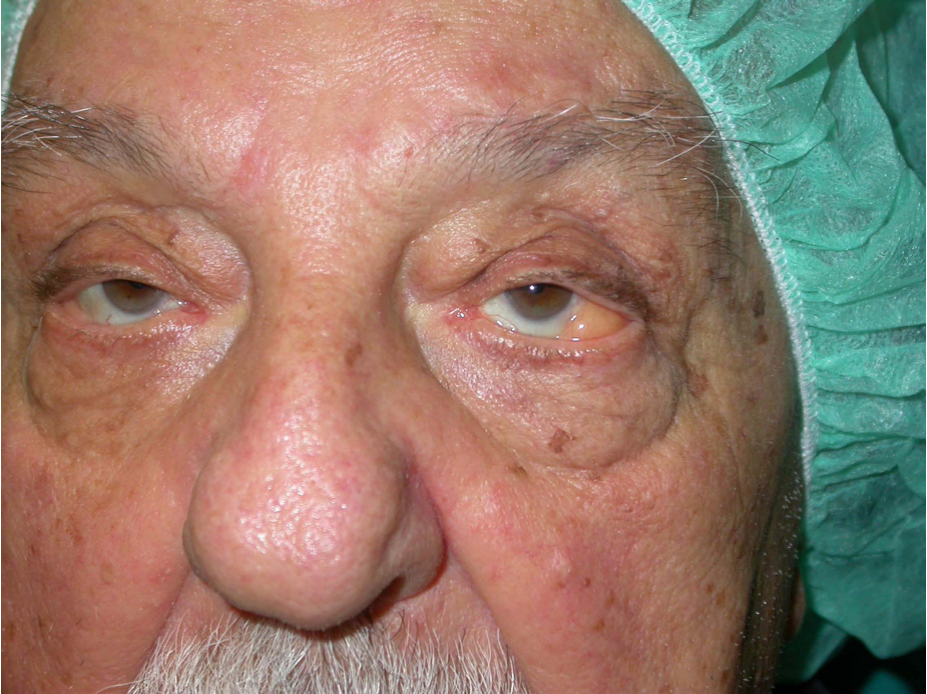
**68 year-old patient with palpebral ptosis and bulging fatty tissue "inside" the eyelid**.

**Figure 2:**
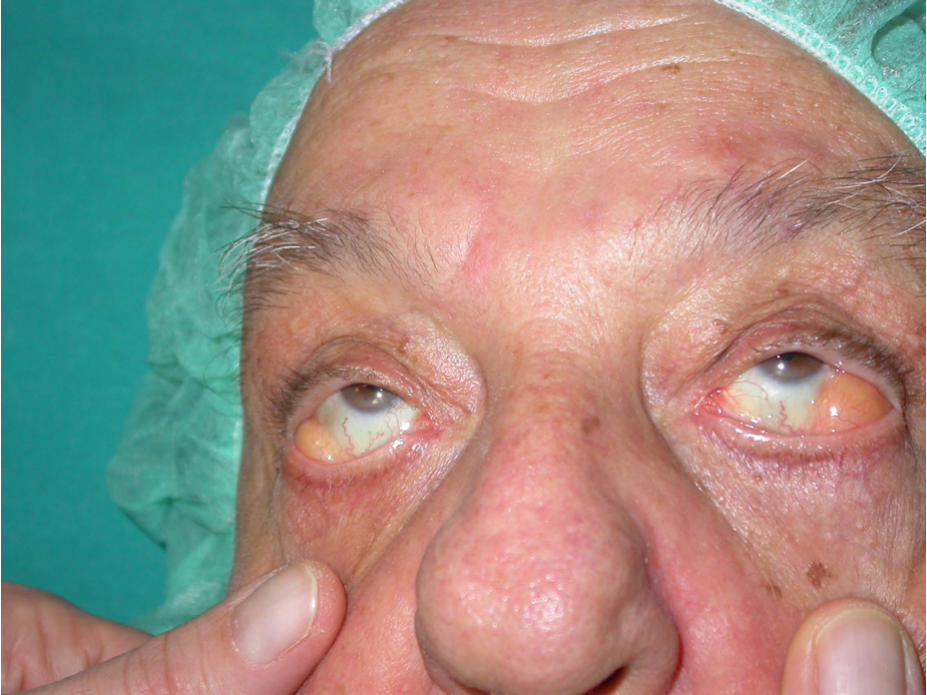
**Evidence of fat in upgaze view**.

This brief report describes the case of a patient who was referred to us for the correction of a second degree bilateral palpebral ptosis that the patient had suffered from for several years and that in recent months had worsened to the point of interfering with vision and who, aside from modest eyelid bags, presented a massive protrusion of "preocular" fatty tissue. This 68 year-old patient did not present other distinguishing abnormalities, aside from intermediate obesity. An examination revealed the presence of fatty tissue, which after having made its way through the conjunctival fornix and partly occupying it, had settled in front of the eyeball, protruding through the margin of the eyelid (Fig. 1), extending completely to the left, and to a lesser degree to the right, although especially evident in upgaze (Fig. 2). In the preoperative phase, along with a surgical procedure to correct the palpebral ptosis, the patient was also offered the option of removing the bulging fatty tissue, which was accepted only because it would be performed through a transconjunctival approach. In fact, despite the indication of classic blepharoplasty through a lower lid incision and, therefore, the possibility of removing excess skin, the patient opted instead only for the removal of the bulging fat. Moreover, the patient even refused a possible cantoplastic intervention to tighten the floppy eyelid, a probable concurrent cause of the patient's abnormal condition. The patient's postoperative results were normal and the patient was extremely satisfied with both the correction of the ptosis and the "rejuvenating" effect of removing the protruding orbital fat in the eyelid.

## Conclusion

We believe that our patient's case is of interest because, even though it is not rare, it is not normally covered in textbooks and specialized articles on periorbitary surgery.

## Competing interests

The authors declare that they have no competing interests.

## Authors' contributions

FI, FMA and RC participated in the surgical treatment and in the follow-up examinations. MT drafted the manuscript and revised the literature sources. MM and GD participated in the follow-up examinations.

ADI revised the literature sources. AMI managed the data collection and contributed to writing the paper. All authors read and approved the final manuscript.

## Consent Statement

Written informed consent was obtained from the patient for publication of this case report and accompanying images. A copy of the written consent is available for review by the Editor-in-Chief of this journal.

## References

[B1] Goldberg RA McCann JD Fiaschetti D Simon GJB What causes eyelid bags? Analysis of 114 consecutive patients Plast Reconstr Surg 2005 115 1395 10.1097/01.PRS.0000157016.49072.61 15809605

